# Obesity: Pollutants May Put on the Pounds

**Published:** 2006-12

**Authors:** Bob Weinhold

Most of the change we think we see in life is due to truths being in and out of favor.

Robert Frost

The Black Cottage

The worldwide obesity epidemic is usually blamed on overeating and underexercising. But limited evidence has suggested a few environmental contaminants may also be playing a role. Now some of the first detailed evidence implicating organotins, a class of persistent compounds containing at least one tin–carbon bond, has been published in the September 2006 *Molecular Endocrinology.* A team of U.S. and Japanese researchers found both *in vitro* and *in vivo* evidence that exposure to a number of organotins, at concentrations typically found in people and wildlife, can contribute to alterations in pathways known to play a key role in excess weight gain, and can lead to significant aberrations in fat cells in mice and frogs.

As in many countries around the world, the percentage of people in the United States who are overweight and obese has been rising sharply in the past 30 years or so. Excess weight is strongly linked with many serious health problems, including heart disease, type 2 diabetes, high blood pressure, and some cancers. Prevention of obesity is by far the best available treatment. Preventing exposures to environmental contaminants over the course of a lifetime, even prior to conception, may be an important part of the battle, if this and other recent papers hold up to scrutiny.

Bruce Blumberg, an assistant professor of developmental and cell biology at the University of California, Irvine, and his colleagues studied organotins partly because of their endocrine-disrupting effects. Growing evidence suggests that some endocrine disruptors (such as bisphenol A and nonylphenol) can play a role in weight gain (although others, such as genistein, can protect against weight gain). Organotins are widespread through their use in boat hull antifouling paints, pesticides, wood preservatives, textiles (as a biocidal agent), plastics, and other products. Many studies have documented adverse health and environmental effects of organotins, from masculinization in some fish to hepatotoxicity in some mammals. Human exposure is largely through consumption of contaminated foods and contact with treated materials.

The team assessed several organotins to gauge their effects on weight gain, and found that a few, particularly tributyltin chloride, were strongly implicated. Others, such as bis(triphenyltin) oxide, dibutyltin, tetrabutyltin, and the dialkyltins, also showed some effects. To varying degrees, *in vitro* and *in vivo* exposures in mice and frogs disrupted the normal function of retinoid X receptor α, β, and γ and/or peroxisome proliferator–activated receptorγ, all of which play key roles in a number of processes related to fat cell differentiation.

Exposure in neonatal mice also led to significant perturbation of signaling pathways and aberrant fat cell formation at several sites, including the liver, testis, and epididymis (where sperm are stored and become mature). *In utero* exposure in mice also led to greater accumulation of fat in several sites after the mice were born. Further, although the birth weight of mouse pups exposed *in utero* tended to be normal, at age 10 weeks the fat content in their epididymis was 20% higher than normal. Aberrant development of fat tissues around the gonads in both males and females also occurred in the frogs. These findings fit with research by others showing that humans can be underweight at birth, but can quickly become overweight, possibly because their fat cell content or function is aberrant.

The Blumberg team’s findings are significant, and may be just the tip of the iceberg, says Paul Cooke, a professor of reproductive biology at the University of Illinois at Urbana–Champaign. Blumberg and colleagues observed that estimates of health effects based on structure–function relationships—typically used to gauge the effects of a group of chemicals—would not have predicted the observed effects of organotins since organotin structures do not resemble those of other compounds that activate retinoid X receptors and peroxisome proliferator–activated receptor γ. According to Cooke, the results therefore suggest that other chemicals that affect various hormone signaling pathways may play a similar role in weight gain.

## Figures and Tables

**Figure:**
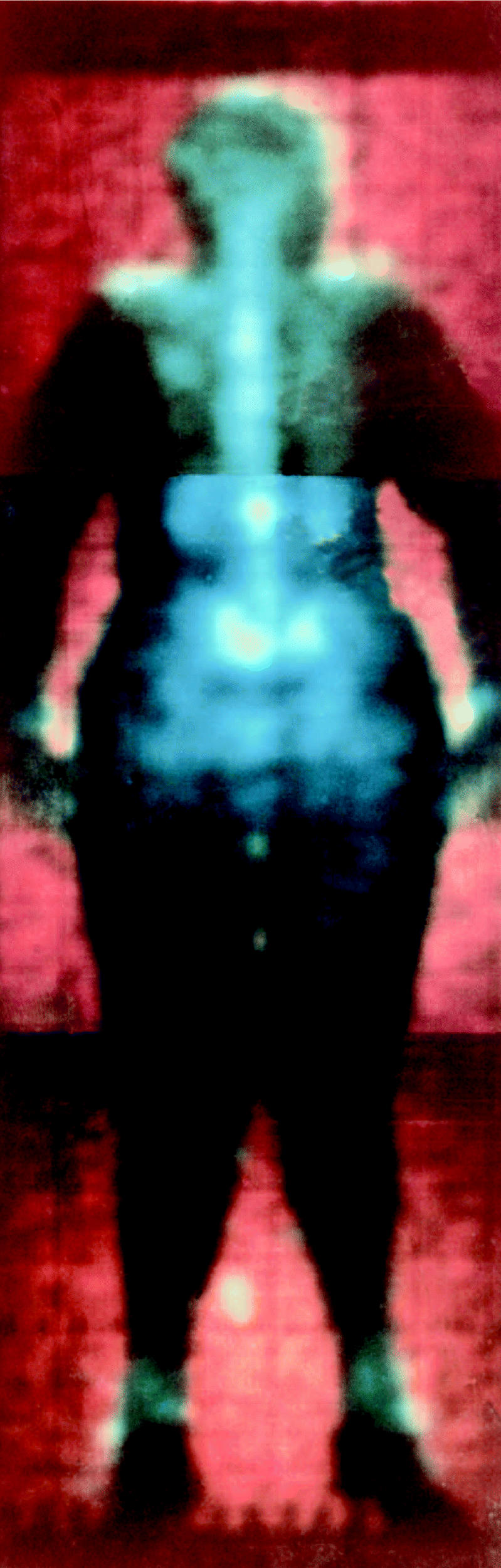
Toxic tally New data suggest exposure to certain pollutants may exacerbate weight gain.

